# Computed Tomographic and Histopathological Characteristics of 13 Equine and 10 Feline Oral and Sinonasal Squamous Cell Carcinomas

**DOI:** 10.3389/fvets.2020.591437

**Published:** 2020-11-23

**Authors:** Carina Strohmayer, Andrea Klang, Sibylle Kneissl

**Affiliations:** ^1^Diagnostic Imaging, University of Veterinary Medicine, Vienna, Austria; ^2^Department of Pathobiology, Institute of Pathology, University of Veterinary Medicine, Vienna, Austria

**Keywords:** cat, computed tomography, histology, horse, malignancy grading, oral and sinonasal squamous cell carcinoma

## Abstract

Squamous cell carcinoma (SCC) is the most common equine sinonasal and feline oral tumour. This study aimed to describe the computed tomographic and histopathological characteristics of equine and feline SCC. Thirteen horses and 10 cats that had been histopathologically diagnosed with oral or sinonasal SCC and had undergone computed tomography (CT) of the head were retrospectively included in the study. CT characteristics of the mass and involved structures were noted. Histological examinations were evaluated according to a human malignancy grading system for oral SCC, which considered four grades of increasing aggressiveness. In horses, the masses were at the levels of the paranasal sinuses (*n* = 8), mandible (*n* = 3), tongue (*n* = 1), and nasal cavity (*n* = 1). In cats, the masses were at the levels of the maxilla (*n* = 4), mandible (*n* = 3), tongue (*n* = 1), and buccal region (*n* = 1) and were diffusely distributed (facial and cranial bones; *n* = 1). Masses in the equine paranasal sinuses showed only mild, solid/laminar, periosteal reactions with variable cortical destruction. However, maxillary lesions in cats showed severe cortical destruction and irregular, amorphous/pumice stone-like, periosteal reactions. CT revealed different SCC phenotypes that were unrelated to the histological grade. For morphologic parameters of the tumour cell population, a variability for the degree of keratinization and number of mitotic cells was noted in horses and cats. Concerning the tumour-host relationship a marked, extensive and deep invasion into the bone in the majority of horses and cats was seen. Most cases in both the horses and cats were categorized as histological grade III (*n* = 8); four horses and one cat were categorized as grade IV, and one horse and one cat were categorized as grade II. In this study, we examined the diagnostic images and corresponding applied human histopathological grading of SCC to further elucidate the correlations between pathology and oral and sinonasal SCC imaging in horses and cats.

## Introduction

Oral and sinonasal squamous cell carcinomas (SCCs) are keratinocyte tumours derived from the stratified squamous epithelium of the mucosa (1). SCCs are thought to be the most common nasal and paranasal tumour in horses (2, 3) and the most common malignant oral tumour in cats, accounting for 60–70% of all feline malignant oral tumours (4, 5). In horses, ~7% of SCCs are oral (6), and in cats, ~85% of SCCs occur in the head region (7). SCCs are aggressive, infiltrative tumours with pre-dilection for the gingiva and mucosa of the maxillary, mandibular, lingual, tonsillar, lip, and buccal regions (5). Because of their location and infiltrative nature, complete excision is often impossible; thus, current oncologic protocols focus on controlling the primary tumour (8).

Computed tomography (CT) is commonly used in human medicine to assess the local extent and regional lymph nodes in head and neck squamous cell carcinoma (HNSCC) (9). However, few case reports have described the CT characteristics of SCC in the equine head and neck region (10, 11). Kowalczyk et al. (10) provided the most detailed imaging description of two horses with SCC in the paranasal sinuses showing a lobulated heterogeneous soft tissue mass with osteolysis and irregular periosteal new bone formation. Several studies have described oral SCC in cats (12–14). Common imaging features in cats include a mass lesion with marked heterogeneous contrast enhancement and osteolysis (12).

Histologically, SCCs are described according to cellular appearance and can be further subclassified (15). In human medicine, detailed grading schemes focus on different aspects of oral SCC (16). Animals and humans share some aspects of the pathogenesis in certain tumours, such as squamous cell carcinoma, and thus the term “one health” has been introduced and discussed recently (17). Better understanding SCC on a cellular level could support better tumour characterization and consequently better individual treatment and prognosis. Evaluating these two most commonly affected companion animal species relative to SCC could determine a wide range of characteristics related to equine and feline HNSCC.

## Materials and Methods

### Specimens

Medical records of horses and cats from 2002 to 2019 were reviewed and were included in the study if the animal had undergone a CT examination of the head region and if histopathology of the oral or sinonasal SCC was available. Table 1 lists the breed, sex, age at time of diagnosis, days between CT and histological sampling, and mode of sample collection. Additionally, the survival times (amount of days between histopathological diagnosis and last day of follow up or euthanasia) for all horses and cats were retrieved from the medical records.

**Table 1:** Information on patients and relevant time points of an equine and feline population diagnosed with oral or sinonasal squamous cell carcinoma.

	**Breed**	**Sex**	**Age at diagnosis (years)**	**Time between computed tomography and histological sampling (days)**	**Mode of sample collection**
Horse 1	Haflinger	f	22	7	N
Horse 2	Warmblood mix	mc	22	3	B
Horse 3	Trakehner	mc	19	12	B
Horse 4	Connemara pony	f	16	6	N
Horse 5	Haflinger	mc	7	1	N
Horse 6	Icelandic horse	f	18	3	N
Horse 7	Trotter	f	8	0	N
Horse 8	Icelandic horse	mc	16	0	N
Horse 9	Shetland pony	f	26	0	N
Horse 10	Warmblood	f	20	0	N
Horse 11	Noric horse	mc	17	4	B
Horse 12	Pony	mc	27	0	N
Horse 13	Trotter	mc	12	6	B
Cat 1	European Shorthair	mc	10	1	B
Cat 2	European Shorthair	fs	5	0	B
Cat 3	Persian cat	fs	14	0	B
Cat 4	European Shorthair	fs	15	0	B
Cat 5	European Shorthair	mc	13	58	N
Cat 6	European Shorthair	mc	15	0	B
Cat 7	European Shorthair	mc	11	0	B
Cat 8	European Shorthair	fs	15	0	N
Cat 9	European Shorthair	fs	13	0	B
Cat 10	European Shorthair	fs	14	15	B

*mc, male castrated; fs, female spayed; f, female; N, necropsy; B, biopsy*.

### Computed Tomography

CT scans of the head from 2002 to 2008 were performed using a single-slice CT scanner (Pace^TM^, General Electric, Milwaukee, WI, USA), and those from 2009 to 2019 were performed using a 16-slice CT scanner (SOMATOM^Ⓡ^ Emotion 16, Siemens Healthcare, Erlangen, Germany). Under general anaesthesia, all horses except one were positioned in dorsal recumbency, and cats were positioned in ventral recumbency. Images were acquired after euthanasia for three horses the same or the following day. For contrast studies, iodinated non-ionic contrast medium (iopamidol, 370 mgI/ml) was administered intravenously at 200–300 mgI/kg in horses and 600 mgI/kg in cats. Three contrast phases were available in two horse and six cats; two contrast phases were available in two cats. The CT images were reviewed with an image analysis programme (JiveX, VISUS Health IT GmbH, Germany, Version 5.2.1.). Two radiologists blinded to the histopathological grading but aware of the diagnosis of squamous cell carcinoma evaluated the following parameters in consensus in a bone window and soft tissue window pre- and post-intravenous contrast medium (when available): the centre of the mass, margination (poor or well-defined), attenuation of the mass (in hounsfield units (HU); a circular region of interest as large as practicable was placed on pre- and post-contrast images), contrast enhancement (homogeneous, heterogeneous, or rim enhancement), size of the mass (Cats: maximum length of the mass in mm on post-contrast images; Horses: sinonasal masses classified as small (less than a third of the size of the maxillary sinus), medium (not extending approximately the size of the maxillary sinus), large (extending the maxillary sinus), oral masses: maximum length of the mass in mm on pre-contrast or if available post-contrast images), number of involved bones, number of involved tissue types (bone, soft tissues, fat, skin), number of involved compartments (oral cavity, pharynx, nasal cavity, nasopharynx, paranasal sinus, orbital region, cranial cavity, or soft tissues), pattern of new bone formation (mild or severe; solid/lamellar, irregular, spiculated, Codman's triangle, or amorphous/pumice stone-like), cortical destruction (mild or severe; permeative and/ or gross), and regional lymph node evaluation. Evaluation of the lymph nodes was performed on CT images in a soft tissue window pre- or, if available, post-administration of contrast medium. Moderate asymmetry to the contralateral side was subjectively assessed. For measurement of the lymph nodes the maximum long axis dimension of the biggest mandibular and medial retropharyngeal lymph node of each side and the maximum perpendicular short axis dimension was noted on transverse CT images. The maximum length of each lymph node was measured on sagittal CT images along the long axis of the lymph nodes. The respective shape of these lymph nodes was described as fusiform, irregular, oval, or round. The number of lymph nodes was noted as: not seen, 1, 2, 3, ≤10, ≥10, ≥20.

### Histopathology

Formalin-fixed, paraffin-embedded tissue samples were retrieved from the archive, prepared on slides and stained with haematoxylin and eosin. One pathologist, who was unaware of the CT findings, reviewed the slides. The samples were evaluated according to a human malignancy grading system by Anneroth et al. (18) for oral SCC considering morphological parameters of the tumour cells and the tumour-host relationship. For all animals, the degree of keratinization, nuclear polymorphism and the average number of mitotic cells per high-power field (HPF) analyzed in 10 HPFs were evaluated as morphologic parameters and hallmarks of malignancy in tumour cell populations. Additionally, tumour-host interactions were considered, including the pattern and stage of invasion and lymphoplasmocytic infiltration. Each of these six parameters were graded from 1 to 4 points (Table 2). The total score was allocated to four grades.

**Table 2:** Malignancy grading system of oral squamous cell carcinoma according to Anneroth et al. (18).

**Histologic grading of malignancy of tumour cell population**				
**Points**				
**Morphologic parameter**	**1**	**2**	**3**	**4**
Degree of keratinization	Highly (>50% of the cells)	Moderate (20–50% of cells)	Minimal (5–20% of cells)	None (0–5% of cells)
Nuclear polymorphism	Little (>75% mature cells)	Moderately abundant (50–75% mature cells)	Abundant (25–50% mature cells)	Extreme (0–25% mature cells)
Number of mitotic cells/high-power field	0–1	2–3	4–5	>5
**Histologic grading of malignancy of tumour-host relationship**				
Pattern of invasion	Pushing, well-delineated infiltrating borders	Infiltrating, solid cords, bands and/or strands	Small groups or cords of infiltrating cells (*n* > 15)	Marked and wide-spread cellular dissociation in small groups of cells (*n* <15) and/or in single cells
Stage of invasion (depth)	Carcinoma *in situ* and/or questionable invasion	Distinct invasion, but involving lamina propria only	Invasion below lamina propria adjacent to muscles, salivary gland tissues and periosteum	Extensive and deep invasion replacing most of the stromal tissue and infiltrating jawbone
Lympho-plasmocytic infiltration	Marked	Moderate	Slight	None

## Results

### Specimens

Twenty-three patients (13 horses and 10 cats) met the inclusion criteria. No animal underwent chemotherapy or radiation therapy. Survival times in horses ranged from 0 to 306 days. Patient owners decided for euthanasia in five horses within the 1st day after CT examination due to extensive involvement of the mass. One horse was euthanized due to poor clinical presentation and cytological diagnosis of squamous cell carcinoma, which was confirmed post mortem by histopathology. Five horses were euthanized 5–10 days after histopathological diagnosis with additional pain medication. Two horses underwent surgical debulking with additional pain medication. One horse was euthanized 70 days and one horse 306 days after histopathological diagnosis. The survival times in cats ranged from 0 to 67 days. Four out of 10 cats were euthanized on the same day of CT or histopathological diagnosis. One cat was euthanized after 4 days following histopathology. The remaining five cats were treated conservatively with antibiotics, anti-inflammatory medication, pain control medication, or a range of combination of those. Of those, two cats were lost to follow on days 0, and one cat each 16 days and 32 days after the histopathological diagnosis. One cat was euthanized due to clinical deterioration 67 days after diagnosis. Table 3 summarizes the differences in CT findings.

**Table 3:** Computed tomography (CT) features of oral or sinonasal squamous cell carcinomas in an equine and feline population.

	**CT scanner (slices)**	**Involved compartments (Number)**	**Involved tissue layers (Number)**	**Involved bones (Number)**	**Periosteal patterns (Number)**	**Centre of the mass(right, left)**	**Size**
Horse 1	multi	4	1	3	1	Paranasal sinuses (right)	large
Horse 2	multi	1	0	0	0	Nasal cavity (left)	small
Horse 3	multi	3	1	2	1	Paranasal sinuses (left)	large
Horse 4	multi	2	2	1	3	Mandible (right)	146 mm
Horse 5	multi	3	3	2	1	Paranasal sinuses (left)	large
Horse 6	multi	3	4	2	2	Paranasal sinuses (right)	medium
Horse 7	single	8	4	7	1	Paranasal sinuses (left)	medium
Horse 8	single	6	4	8	1	Paranasal sinuses (left)	large
Horse 9	multi	3	2	1	3	Mandible (right)	81 mm
Horse 10	multi	2	2	1	2	Soft tissue–tongue (left)	75 mm
Horse 11	multi	3	3	2	1	Paranasal sinuses (right)	large
Horse 12	multi	2	2	1	2	Mandible (right)	112 mm
Horse 13	multi	4	4	3	1	Paranasal sinuses (left)	large
Cat 1	multi	5	4	4	1	Maxilla at the level of the orbit (left)	32 mm
Cat 2	single	4	4	2	1	Mandible (left)	58 mm
Cat 3	single	5	4	10	1	Diffuse (facial and cranial bones) (left)	45 mm
Cat 4	multi	3	3	4	2	Mandible (right)	ne
Cat 5	multi	2	3	0	0	Soft tissue–buccal (left)	26 mm
Cat 6	multi	3	2	0	0	Soft tissue–tongue (right)	34 mm
Cat 7	multi	6	4	8	1	Maxilla at the level of the orbit (right)	40 mm
Cat 8	multi	2	4	2	1	Mandible (right, left)	ne
Cat 9	multi	5	4	5	1	Maxilla at the level of the orbit (right)	35 mm
Cat 10	multi	6	4	10	1	Maxilla at the level of the orbit (left)	50 mm

*Compartments (oral cavity, pharynx, nasal cavity, nasopharynx, paranasal sinus, orbital region, cranial cavity, soft tissues); tissue layers (bone, soft tissues, fat, skin); periosteal patterns (solid/lamellar, irregular, spiculated, Codman's triangle, amorphous/pumice stone-like). Ne, not evaluable*.

### Computed Tomography

#### Tumour Characteristics

In horses, the centre of the masses occurred at the paranasal sinus, mandible, tongue, and nasal cavity levels, with the centre at the paranasal sinus in eight out of 13 cases. Of these eight cases, six had soft tissue attenuation filling almost the complete ipsilateral rostral and caudal maxillary sinus, dorsal conchal sinus, ventral conchal sinus, while the conchofrontal and sphenopalatine sinus showed different amount of filling. In one case, the soft tissue mass also affected the contralateral side. In two of the eight horses, one showed nodular masses involving a third of the ipsilateral rostral maxillary sinus and less than a third of the conchofrontal sinus adjacent to the rostral maxillary sinus. In the second horse, the complete rostral maxillary sinus and less than one third of the conchofrontal sinus were affected. In three of the 13 horses, masses were localized at the mandible with varying soft tissue mass extensions. One equine lingual SCC had concurrent involvement of the ipsilateral stylohyoid bone. The fewest changes were noted in one horse with mild unilateral narrowing of the common and ventral nasal meatus by small nodular soft tissue lesions along the nasal septum. In two horses contrast medium was applied and heterogeneous contrast enhancement could be detected, however the masses were poorly defined. The margination of the masses could not be evaluated in 11/13 horses due to lack of contrast medium application. Six out of eight masses in the paranasal sinus were classified as large and mean maximum length of oral masses was 103.5 ± 32.6 mm (range, 75–146 mm). The mean pre-contrast attenuation values of the masses were 41.5 ± 7.7 HU (range, 30–55.1 HU). The maximum attenuation values of the two horses after contrast medium application were 64.2 and 54.2 HU.

In cats, the centre of the masses occurred at the maxilla, mandible, tongue, and buccal levels, and in one case, the tumour displayed diffuse involvement of the facial and cranial bones. In all cases, the masses were poorly marginated. Five out of eight cases showed a rim enhancement and three out of eight cases showed a heterogeneous enhancement pattern of the masses. Four of the 10 cats had a mass centred at the maxilla. Degrees of exophthalmus varied depending on mass size. Three of the 10 cats showed the centre of the mass at the mandible, two had only a soft tissue mass, one in the buccal region, and one affecting the tongue. One cat with diffuse disease showed a poorly marginated soft tissue mass along the ventral aspect of the nasal cavity, unilateral maxilla, ipsilateral buccal region, with intracranial extension and affecting the ipsilateral mandibula as well. The mean maximum length of the masses was 40 ± 10.5 mm (range, 26–58 mm) post-contrast. The masses showed a mean attenuation value of 60.6 ± 15.6 HU (range, 46.6–90.8 HU) on pre-contrast images. After contrast medium application the mean attenuation values were 106.8 ± 32.6 HU (range, 69.5–163.8 HU).

#### Bone Changes

In eight equine patients tumour masses were at the paranasal sinus level. Seven of those eight horses had associated mild, solid-to-laminar periosteal reactions. One horse had a severe solid-to-laminar periosteal reaction and an additional mild irregular periosteal reaction. All cases showed some degree of cortical destruction. Cases with gross destruction were severe; cases with permeative destruction were mild. Mandibular masses in two horses showed severe periosteal reaction and cortical destruction; these periosteal reactions were solid/lamellar and spiculated, with a Codman's triangle and amorphous periosteal reactions. One horse presented only a mild, solid/lamellar, periosteal reaction with a Codman's triangle and severe, gross mandibular cortical destruction. One equine patient with a lingual mass showed osseous changes of the ipsilateral stylohyoid bone, including severe, gross cortical destruction, with mild, irregular, periosteal reactions and a Codman's triangle.

The feline maxillary tumours showed severe, gross, permeative destruction. Periosteal reactions were severe, irregular, and amorphous/pumice stone-like for the maxillary lesions. Mandible-associated masses in the cats showed different periosteal reactions (solid/lamellar, irregular and spiculated, or amorphous/pumice stone-like). Two cats showed mild, permeative cortical and severe, gross cortical destruction, while in one case both permeative and gross cortical destruction were severe. One cat had a diffuse soft tissue mass involving the facial and cranial bones, which showed mainly gross cortical destruction and mild, irregular periosteal reaction.

#### Involved Compartments

Ten of the 13 horses had two to four compartments involved. The most common involved compartments were nasal cavity, paranasal sinuses, and soft tissues. Two-thirds of the cases (8/13) showed two and four affected tissue layers.

Cats displayed between two and six involved compartments, which were almost equally distributed, and most cats (9/10) revealed three and four affected tissue layers.

#### Lymph Nodes

**Figure 1:**
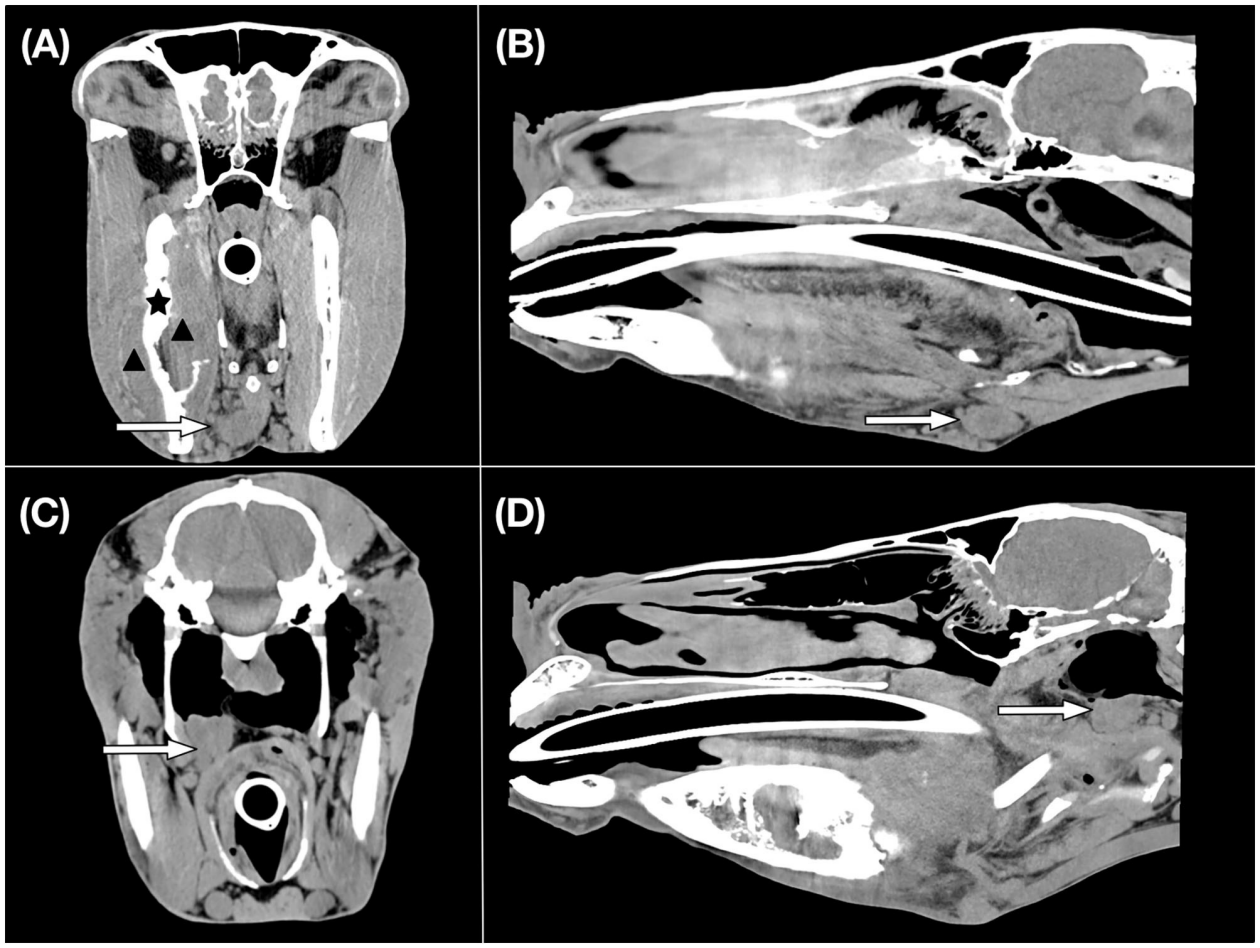
CT images of an equine patient diagnosed with an oral squamous cell carcinoma centred at the right mandible. Transverse **(A)** and sagittal **(B)** CT images in a soft tissue window show an enlarged right mandibular lymph node which is indicated by a white arrow. Histopathological examination revealed evidence of metastasis. A black asterisk illustrates the site of osseous changes of the right mandible. The black arrowheads indicate the region of the soft tissue mass. Transverse **(C)** and sagittal **(D)** CT images in a soft tissue window display an enlarged right medial retropharyngeal lymph node shown by a white arrow.

For regional lymph nodes in the horses, 20 of 26 mandibular and 14 of 26 medial retropharyngeal lymph nodes were available for interpretation. Eight ipsilateral and one contralateral mandibular lymph nodes and five ipsilateral and two contralateral medial retropharyngeal lymph nodes were subjectively enlarged in nine horses. The mean maximum length × short axis _trans_ × long axis _trans_ of the largest right and left mandibular lymph node was 28.5 ×12.8 ×18.8 mm and 35.0 ×16.5 ×20.7 mm, respectively. The mean maximum length × short axis _trans_ × long axis _trans_ of the largest right and left medial retropharyngeal lymph was 36.1 ×21.4 ×26.2 mm and 32.9 ×17.3 ×25.9 mm, respectively. An example of the CT appearance of regional lymph node enlargement is shown in Figure 1.

Twenty of 20 groups of mandibular lymph nodes and 20 of 20 medial retropharyngeal regional lymph nodes were available for interpretation. Four ipsilateral mandibular and three ipsilateral medial retropharyngeal lymph nodes were subjectively enlarged in four cats. In cats, the mean maximum length × short axis _trans_ × long axis _trans_ of the largest right and left mandibular lymph node was 9.5 ×3.3 ×6.5 mm and 8.9 ×3.5 ×5.8 mm, respectively. The mean maximum length × short axis _trans_ × long axis _trans_ of the right and left medial retropharyngeal lymph nodes was 16.7 ×3.6 ×10.5 mm and 15.6 ×3.9 ×10.3 mm, respectively.

Table 4 lists all individual lymph node parameters for each patient.

**Table 4:** Features of mandibular and medial retropharyngeal lymph nodes in an equine and feline population diagnosed with oral or sinonasal squamous cell carcinoma.

	**Mandibular lymph nodes (mm) right; left**					**Medial retropharyngeal lymph nodes (mm) right; left**				
	**Long axis**	**Short axis**	**Length**	**Shape**	**Number**	**Long axis**	**Short axis**	**Length**	**Shape**	**Number**
Horse 1^*^	na	na	na	na	na	na	na	na	na	na
Horse 2	15.2; 16.2	8.9; 11.2	30.4; 25.8	i; i	≥20; ≥20	14.2; 17.3	9.1; 10.2	34.0; 29.8	o; o	≥10; ≥10
Horse 3^*^	15.8; 26.0	12.0; 21.8	17.6; 60.7	o; i	≥20; ≥20	na	na	na	na	na
Horse 4^*^	42.9; 17.9	24.8; 15.3	53.6; 40.5	i; o	≥20; ≥20	31.8; 9.9	27.6; 5.9	39.4; 11.1	i; o	≥10; ≥10
Horse 5^*^	7.4; 39.8	6.3; 29.8	15.5; 55.7	o; i	≥20; ≥20	na	na	na	na	na
Horse 6^*^	16.7; 12.8	12.1; 5.0	27.8; 29.1	i; o	≥20; ≥20	na	na	na	na	na
Horse 7^*^	na	na	na	na	na	na	na	na	na	na
Horse 8^*^	8.7; 21.2	7.3; 19.7	16.1; 34.0	o; i	≥10; ≥10	ne	ne	ne	ne	ne
Horse 9^*^	22.5; 6.7	13.4; 6.6	29.9; 7.6	i; o	≤ 10; ≤ 10	41.9; ne	40.9; ne	56.3; ne	i; ne	1; ne
Horse 10	12.4; 15.3	8.9; 14.6	27.0; 27.7	i; i	≥20; ≥20	28.8; 29.7	17.0;18.7	31.2; 29.2	i; i	≥10; ≥10
Horse 11^*^	na	na	na	na	na	na	na	na	na	na
Horse 12^*^	27.6; 23.6	22.1; 15.9	35.8; 31.5	i;i	≤ 10; ≤ 10	ne; 38.3	ne; 28.2	ne; 58.1	ne; i	ne; ≤ 10
Horse 13^*^	18.9; 27.6	12.9; 24.6	31.3; 37.8	i; i	≥20; ≥20	14.4; 34.6	12.5; 23.7	19.7; 36.6	o; i	≤ 10; ≤ 10
Cat 1	8.2; 8.0	3.0; 6.4	12.9; 15.2	o; r	1; 2	9.0; 11.8	3.3; 3.5	16.5; 16.4	f; f	1; 1
Cat 2	8.6; ne	5.5; ne	10.8; ne	r; ne	2; ne	12.3; 15.2	4.0; 7.3	17.6; 18.0	f; o	1; 1
Cat 3	2.9; 4.7	1.6; 2.8	6.0; 6.7	o; o	2; 2	7.1; 7.7	2.0; 2.1	9.9; 9.8	f; f	1; 1
Cat 4^*^	ne; 5.0	ne; 3.1	ne; 9.3	ne o	ne; 2	9.9; 10.0	3.8; 4.2	13.3; 13.8	o; o	1; 1
Cat 5	9.1; 10.3	3.7; 4.9	12.2; 11.5	r; r	2; 2	11.4; 12.5	4.0; 4.4	25.7; 24.8	f; f	1; 1
Cat 6	4.0; 3.4	3.4; 1.9	5.1; 6.3	r; o	2; 2	15.9; 10.4	4.6; 3.3	19.0; 17.3	o; f	1; 1
Cat 7	7.5; 6.8	3.3; 4.7	10.0; 7.6	r; o	2; 2	11.0; 9.1	4.0; 4.2	19.4; 18.0	f; f	1; 1
Cat 8^*^	4.5; 4.9	2.2; 2.6	7.3; 8.6	o; o	2; 2	7.7; 6.6	3.5; 3.7	11.3; 9.1	f; f	1; 1
Cat 9	6.9; 3.4	3.4; 1.6	11.9; 5.6	r; o	2; 2	10.5; 9.0	3.1; 2.1	17.8; 12.8	f; f	1; 1
Cat 10	4.3; 4.8	2.9; 2.1	8.5; 7.6	o; o	2; 3	10.6; 10.0	4.6; 3.6	18.6; 14.4	o; o	1; 1

*Long and short axis are measures on transverse CT images. The maximum length is measured on sagittal CT images along the long axis of the lymph nodes. r, round; o, oval; f, fusiform; b, bilobed*;

* *no contrast medium application; na, not available; ne, not evaluable*.

### Histopathology

#### Tumour

The majority of horses (8/13) was categorized as grade III, four cases as grade IV and one case was grade II. The degree of keratinization was distributed relatively unequal within the study population, with increasing numbers from highly keratinized cells to none keratinization. The latter group comprised almost 40 % of the study population (5/13). Four horses showed a minimal (4/13) and three a moderate degree of keratinization (4/13), whereas only one case displayed highly keratinization. Also, nuclear polymorphism and mitotic cells were quite inconsistent ranging from moderately abundant (4/13), abundant (3/13) to extreme polymorphism (6/13) and zero to more than five mitotic cells per HPF. In most horses (12/13) the tumour turned out to be highly invasive with marked and widespread cellular dissociation in small groups of cells and extensive and deep invasion. Lymphoplasmocytic infiltration was ranging between moderate to none. Eight of 10 cats were categorized as grade III. Out of them two were categorized at least grade III because the small size of the tissue sample did not allow to evaluate a total of 10 HPF for counting of mitotic cells. Each one case was designated as grade IV and II. Regarding the morphologic evaluation of the tumour cell population the degree of keratinization was quite variable ranging between moderate to none. Nuclear polymorphism was quite consistent with moderately abundant to abundant and the number of mitotic cells was quite variable lying between zero and five per HPF. Analysis of tumour-host relationship yielded a marked and widespread pattern of invasion in small groups of cells (10/10), an extensive and deep infiltration into the bone replacing most of the stromal tissue (6/10) in the majority of cats and quite variable none to marked lymphoplasmocytic infiltration. The individual scores for horses and cats are noted in Table 5.

**Table 5:** Malignancy grading scores according to Anneroth et al. (18) in an equine and feline population diagnosed with oral or sinonasal squamous cell carcinoma.

	**Degree of keratinization**	**Nuclear polymorphism**	**Number of mitotic cells/high-power field**	**Pattern of invasion**	**Stage of invasion (depth)**	**Lympho-plasmocytic infiltration**	**Total score**	**Grade**
Horse 1	4	4	4	4	4	3	23	IV
Horse 2	2	2	3	3	4	2	16	III
Horse 3	4	4	4	4	4	3	23	IV
Horse 4	4	3	4	4	4	2	21	IV
Horse 5	4	4	2	4	4	3	21	IV
Horse 6	2	2	1	4	4	2	15	II
Horse 7	3	4	2	4	4	2	19	III
Horse 8	4	4	2	4	4	2	20	III
Horse 9	3	3	3	4	4	2	19	III
Horse 10	1	2	2	4	4	4	17	III
Horse 11	3	4	4	4	3	2	20	III
Horse 12	3	3	3	4	4	2	19	III
Horse 13	2	2	4	4	4	3	19	III
Cat 1	3	2	2	4	4	2	17	III
Cat 2	4	2	3	4	3	3	19	III
Cat 3	3	2	1^*^	4	3	4	≥17	III
Cat 4	3	3	1^*^	4	4	3	≥18	≥III
Cat 5	2	2	3	4	3	2	16	III
Cat 6	2	2	1	4	3	2	14	II
Cat 7	3	2	2	4	4	3	18	III
Cat 8	4	3	3	4	4	4	22	IV
Cat 9	3	3	2	4	4	1	17	III
Cat 10	3	2	1^*^	4	4	4	≥18	≥III

* *Tumour did not extend towards the margins of 10 high-power fields. Grade I = 5–10 points, Grade II = 11–15 points, Grade III = 16–20 points, Grade IV = >20 points*.

#### Lymph Nodes

Eight horses with enlarged lymph nodes were necropsied within 1 week after CT, except for one. Two metastatic mandibular and two metastatic medial retropharyngeal lymph nodes were detected among four horses. One horse with subjectively normal lymph nodes on CT images, showed metastasis to the left mandibular lymph node with cytology, however in this horse no histopathology of the lymph node or necropsy was performed.

Histopathological examination was performed in one cat with enlarged lymph nodes revealing metastasis of the ipsilateral mandibular lymph node.

## Discussion

In the current study, we applied Anneroth's classification for cats and adopted this scheme to the best of our knowledge for its first-time use in grading equine oral and sinonasal SCC. Using Anneroth's grading system, eight horses (61%) and eight cats (80%) were categorized as grade III. For both horses and cats, invasion pattern and stage parameters predominantly received the most points, reflecting the intensive, histological infiltrative nature of this tumour. Whereas, morphologic parameter of the tumour cell population including degree of keratinization and number of mitotic cells were quite variable in horses and cats, the evaluation of the tumour-host relationship yielded a marked and widespread as well as extensive and deep invasion into the bone in the majority of horses and cats. Regarding the proliferation of tumour cells it seems that in the majority of horses (61%) the number of mitotic cells was generally higher with up to >5 mitoses/HPF (5/13) and between four and five mitosis (3/13) when compared with cats. In the latter one, mitotic cells in general were lower and relatively evenly distributed within the study population, ranging between zero and five mitoses/HPF. However, it has to be mentioned that in three cases the sample size was too small to evaluate a total of 10 HPFs, which could alter an exact categorization at least in two cases.

**Figure 2:**
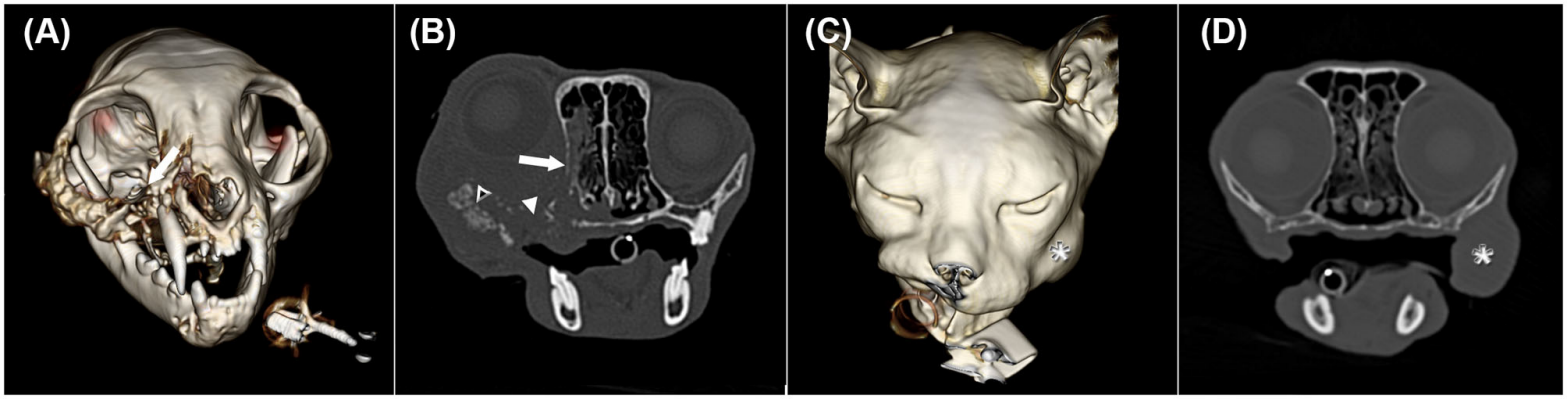
Three-dimensional surface models **(A,C)** and transverse CT images in a bone window **(B,D)** of two cats with oral squamous cell carcinoma showing different CT phenotypes despite having the same histological grade. **(A)** and **(B)** illustrate cat 7 with the centre of the mass at the right maxillar level (white arrow) shown in image A. In image B, severe permeative (white arrow) and gross cortical (white arrowhead) destruction and amorphous/pumice stone-like (black arrowhead with a white frame) periosteal reactions are depicted. The soft tissue mass extends into the ipsilateral oral and nasal cavity, causing ipsilateral exophthalmus. **(C)** and **(D)** represent cat 5 with a mass lesion (white asterisk) at the left buccal level without osseous changes.

**Figure 3:**
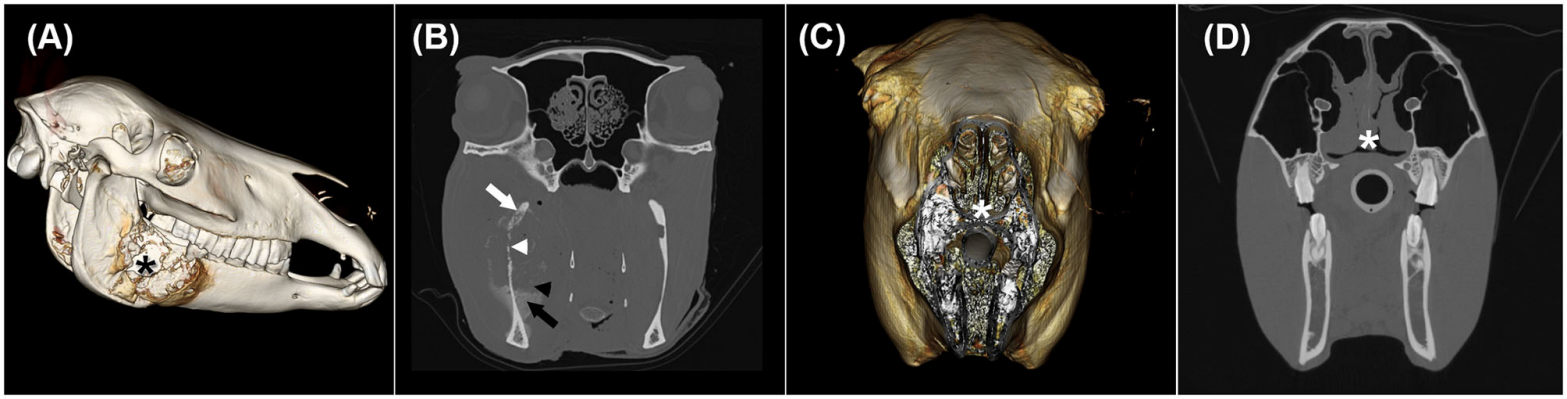
Three-dimensional surface models **(A,C)** and transverse CT images in a bone window **(B,D)** of two horses with oral and nasal squamous cell carcinoma showing different CT phenotypes despite having the same histological grade. **(A)** and **(B)** illustrate horse 9 with a mass lesion affecting the right mandible (black asterisk) with severe permeative (white arrow) and gross cortical (white arrowhead) destruction as well as solid/lamellar (black arrow) and amorphous/pumice stone-like (black arrowhead) periosteal reactions. **(C)** and **(D)** display horse 2 with an irregularly thickened nasal septum (white asterisk).

Most tumours were grade III, and an interesting trend of phenotypic variation within and between the two species was seen on CT images (Figures 2, 3). Consistent with a previous study on felines, similarities in the masses included poor definition, rim and heterogeneous contrast enhancement, and maxillary, mandibular, buccal, and lingual locations (12). Potential patterns of osseous changes also occurred relative to the location and species. In cats, similar features in the maxillary region comprised severe osteolysis and similar periosteal reactions, including irregular and amorphous/pumice stone-like features. In equines, most masses (8/13) were in the paranasal sinuses, which is reported to be more common than in the nasal cavity and particularly frequent in the maxillary sinus. However, whether the mass originates from the sinus or the oral cavity cannot be determined in all cases (3). On CT images, the mass lesions of the paranasal sinuses predominantly showed mild periosteal reactions in the form of a solid-to-lamellar pattern and thus differed from the masses of the maxillary region in cats. Mandible-associated masses showed the most varied periosteal reactions and osteolysis both within species and between species. Cats with masses in the lingual and buccal region presented no osseous changes; however, one horse presented osseous changes affecting the adjacent stylohyoid bone, which indicated local infiltration. Unfortunately, the study population was too small to evaluate a statistically significant correlation between the histological grading system and CT findings.

In addition to primary lesions, regional lymph nodes are assessed in full diagnostic work-ups. The mandibular and medial retropharyngeal lymph nodes are the major lymph nodes draining the oral and nasal cavity (19). In the present study we noted up to 20 mandibular and 10 medial retropharyngeal lymph nodes on soft tissue CT images on each side in horses. Interestingly, a big proportion of the lymph nodes were even smaller than 5 mm, however in addition to the largest lymph nodes measured there were multiple lymph nodes in between in the smallest and largest lymph nodes. To the authors' knowledge no detailed CT descriptions of equine head and neck lymph nodes have been published. According to an anatomical reference, 35–75 densely packed mandibular lymph nodes which can be located along a 16 cm region are reported. For the medial retropharyngeal lymph nodes it has been noted that 20–30 loosely arranged lymph nodes can be present (19).

In cats, the mean maximum length × short axis _trans_ × long axis _trans_ of the largest right and left mandibular lymph node and medial retropharyngeal lymph nodes within ranges of previously described dimensions. There are few studies investigating CT features of feline medial retropharyngeal lymph nodes. One study is focusing on CT measurements of medial retropharyngeal lymph nodes in clinically healthy cats reporting the mean length × rostral height × rostral width dimensions of medial retropharyngeal lymph nodes to be 20.7 ×13.1 ×4.7 mm with the maximum dimensions of ~32 ×20 ×7 mm (20). The same authors published a study comparing medial retropharyngeal lymph node features of cats with nasal neoplasia and rhinitides and found significant association with neoplasia and medial retropharyngeal asymmetry (21). There is limited information about CT characteristics of the feline mandibular lymph nodes. Previously the mean length × height × width dimensions of the right medial and lateral (11.04 ×2.87 ×5.71 mm; 10.86 ×3.22 ×6.53 mm) and left medial and lateral mandibular lymph nodes (11.35 ×2.84 ×5.40 mm; 11.32 ×3.43 ×6.88 mm) of healthy cats have been reported, respectively (22). Gendler et al. (12) described a mean ± SD maximum width of mandibular and medial retropharyngeal lymph nodes of 4.1 ± 1.9 mm (range, 1.5–8.6 mm) and 5.3 ± 1.5 mm (range, 2–8.4 mm), respectively in cats with oral SCC.

In the current study the terms long and short axis were used for the measurements on transverse CT images. While the long and short axis corresponded to the width and height in mandibular lymph nodes and to the height and width in medial retropharyngeal lymph nodes, respectively as described in cats, especially in equine mandibular lymph nodes width and height were not straight forward identified.

The presence of lymph node enlargement was first decided on comparison to the contralateral side. The subjectively enlarged lymph nodes corresponded to the side of the SCC in most of the cases. If no asymmetry is detected, unfortunately bilateral enlargement could not be excluded completely. Measurements of the maximum three dimensions of the largest lymph nodes of each side were made, only in one cat the width of a medial retropharyngeal lymph node was reaching the upper limit for what has been described in healthy cats (20). Unfortunately, histopathological examination of enlarged medial retropharyngeal lymph nodes in cats was not performed. Concerning oral SCC in cats, Gendler et al. (12) reported that the mean ± SD maximum width of mandibular lymph nodes measured on CT images in patients with cytologic evidence of metastasis (5.3 ± 2.1 mm) was not significantly different from the mean maximum width of mandibular lymph nodes in patients without metastatic disease (3.9 ± 1.7 mm).

In the current study few metastatic lymph nodes were detected, accounting for two of nine mandibular and two of seven medial retropharyngeal metastases of enlarged lymph nodes in horses. Equine SCC metastasis is rare, and without a histological examination, enlarged lymph nodes could also result from primary inflammation unrelated to the tumour or could be concurrent inflammation due to secondary tumour infection (3). In the present study, five cats showed subjectively enlarged lymph nodes, but only one lymph node sample was available, which showed mandibular metastasis. Previously, the prevalence of mandibular lymph node metastasis was reported to be 31% in cats. Treating metastasis may be of value in cases where long-term control of the primary tumour is feasible (23). Our results need to be cautiously interpreted since this study was a retrospective study and histopathology of the lymph nodes were only available in a proportion of the cases.

The survival times in horses ranged from 0 to 306 days in the present study. The two horses of this study, who received surgical debulking of masses in the paranasal masses had survival times of 70 days and 306 days. There is little information about the survival time of head and neck SCC in horses. A case report described an 18 year-old American Saddlebred stallion with a SCC at the base of the tongue who received palliative radiation therapy and after initial clinical improvement was euthanized 7 weeks after (24). In another case report, partial excision of the incisive bone was performed in a 27-year-old Arabian stallion with a large oral SCC and showed no gross evidence of recurrence or metastasis 5 months later (6). In the present study the survival times in cats ranged from 0 to 67 days with six of 10 cats being euthanized or lost to follow up on the day of diagnosis. Two previous studies assessed the survival time in cats with oral squamous cell carcinoma of 14–251 days (median 91 days) (23) and 14–1,020 days (median 60 days) (12), with most of the cats being part of a chemotherapeutic trial (12, 23). In another study including cats without radiation therapy or chemotherapy the overall median survival time was 44 days (95% confidence interval (CI): 31–79) (25). The survival times are variable and unfortunately the diagnosis is often achieved at an already progressed disease stage in which local disease control is the main goal. Therefore, with growing knowledge about the characteristics of this tumour, and more precise prognosis for each treatment option, the owners could be provided with more information for decision making.

Histopathological evaluation is part of the diagnostic work-up for equine and feline SCC. Histological grading of equine SCC is generally based on the descriptions of Schuh (26). Schuh's system grades the oral, pharyngeal and nasal mucosa according to morphological cellular characteristics, specifically the number of keratin “pearls,” the number of cells showing keratinization and the presence of intercellular bridges (maximum, moderate, poorly differentiated, and anaplastic variants). For three grades, the amounts of differentiation and keratinization decrease as the grade increases. Grade III additionally describes some atypical mitotic figures (27). To date, more feline than equine studies have addressed the biological background of oral SCC. This is partly because cats are compatible models for research on human head and neck SCC owing to their similar tumour behaviours and response to therapy (28). In human medicine, multiple histological classifications have been used to predict disease behaviour (29). Generally reported histological subtypes in human medicine include conventional SCC (further graded as well-, moderately or poorly differentiated), verrucous carcinoma, basaloid SCC, and other variants (30). Among these classifications, Anneroth's scheme is a multifactorial and detailed human malignancy grading system for oral SCC considering tumour cell populations and tumour-host relationships (18). Akhter et al. (16) investigated Anneroth's classification for human oral SCC compared with other classification systems and concluded that Anneroth's classification can be used as a valuable diagnostic factor. In veterinary medicine, papers on feline oral SCC also used Anneroth's classification (8, 31, 32). Sparger et al. (33) graded feline oral SCC according to human and canine schemes (34, 35). Because of a detailed morphological assessment that included the tumour-host relationship used in human medicine we decided therefore to apply this classification to our study populations.

In humans, HNSCC is known for its heterogenous biological behaviour, thus, HNSCC research focuses on elucidating the genetic background to better understand tumour-specific characteristics (36). Tumours of the same histological type can show various phenotypes, making this tumour challenging to treat (37); however, similarities exist between human and feline oral SCC, thus offering potential options for using cats as animal models (38). Many studies in human medicine are addressing the association between human papillomavirus infection and HNSCC (39). In cats, accumulating studies are also focusing on molecular biology and the relationship with papillomavirus infection (8, 33, 40). Studies have also evaluated using cats as animal models for studying emerging targets in human cancer therapy (41).

In humans, diagnostic imaging shows high variation in the primary tumour locations and degree of involvement of the surrounding structures (42). In addition, the grade of the histological differentiation does not appear to strongly affect predicting the presence or extension of bone involvement (43). Likewise, in cats, oral SCCs can reportedly present as severely aggressive tumours despite being well-differentiated histologically (1). A feline study showed that the lingual location was associated with younger cats, suggesting a possible different pathogenesis, influenced by the epithelial susceptibility to develop SCC (44). In humans, the location seems crucial for the prognosis since the tongue, soft palate, and floor of the mouth present the worst prognoses (45). The squamous epithelium of the oropharynx develops from the endoderm and shows a higher ability to form poorly differentiated carcinomas. Apart from the tumour origin, the pathways by which the tumours spread, i.e., by direct extension over mucosal surfaces, muscle and bone or by lymphatic drainage and extension along neurovascular bundles, must also be considered (42). Because cats and humans have similar bone-invasive phenotypes, cat are recognized as models for characterizing bone resorption in human SCC. Feline oral SCC cells can stimulate osteoclastic bone resorption corresponding to parathyroid hormone related-protein expression (46). A large feline study revealed that bone invasion was most commonly seen in gingival-associated SCC, most likely due to the close anatomical relationship of the gingiva to the bone. New bone formation in the form of periosteal reactions and metaplastic bone as well as the ability of neoplastic cells to directly adhere to the bone matrix have also been described (44). Periosteal reactions invade the bone in human SCC at a frequency of 11% (47). In the current study, eight out of 10 cats and 12/13 horses showed periosteal new bone formation. This high periosteal reaction percentage likely occurred because cats and horses present later in the disease process showing large tumour masses and being more locally aggressive at that stage compared with humans (28). Periosteal reactions occur because of tumour displacement and infiltration. In human medicine, a CT study on periosteal new bone formation in the jaw found a spicule pattern in 61% of malignant tumours, in addition to parallel, irregular and Codman's triangle patterns (47). Those periosteal reactions were also found in the present study. Why severe, irregular and amorphous/pumice stone-like periosteal reactions were common in the maxillary region of cats, and solid-to-lamellar periosteal reactions were seen at the paranasal sinus level in horses is unknown. Different subtypes may be associated with varying numbers of osteoproliferative mediators. Larger study populations are needed to investigate whether specific osseous changes are statistically significant to an anatomical location.

This study had some limitations relative to its retrospective nature. The small sample size prevented statistical analysis. Furthermore, histological evaluations depend on the sample size and location; therefore, biased interpretations can result. As larger tissue samples obtained by necropsy provide better survey of histological findings we generally preferred to use necropsy samples instead of biopsy material as far as possible, however necropsy was only performed in two of 10 cats (the other cats were lost to follow up or were taken home by owners), and was mainly performed in horses. Histopathological samples were acquired by clinicians according to possibilities of biopsy collection, respectively. As this was a retrospective study, an exact correlation between the histological sample location and the respective location on the CT images, which would have helped better elucidate the findings, could not be determined. Some limitations were related to the CT protocols. Because no horse with a mass lesion in the paranasal sinuses received intravenous contrast medium, the proper extent of the mass could not be delineated, and concurrent loculated fluid of varying compositions may have been a contributing factor. Besides, the regional lymph nodes were inconsistently imaged in the horses, and lymph node samples were unavailable in a proportion of animals. Since except for two cases, for all other horses only pre-contrast CT images were available and lymph nodes often presented with an overall irregular shape, it cannot be ensured that this irregular shape resulted from multiple lymph nodes in close proximity to each other and not a single lymph node. Thus, lymph node-related findings must be cautiously interpreted and further studies with healthy controls are warranted. Two horses and two cats were examined with a single-slice CT scanner, which has a lower temporal and contrast resolution in comparison to a multi-slice CT scanner. Thus, the shape of the periosteal reaction or the existence of permeative cortical destruction could have been misinterpreted. The time between CT and histological examination was <7 days in most animals. However, delays of 58 and 15 days occurred between these two diagnostic modalities in two cats, and a 12-day delay occurred in one horse. Whether this time difference affected the morphological changes is unknown. Unfortunately, no studies have documented morphological follow-up. Therefore, the degrees of osseous changes may be partly related to a more progressed disease.

In conclusion, the current study showed that equine and feline oral and sinonasal SCCs presented different phenotypic tendencies on CT scans while also presenting similar histological grades. These findings add diagnostic imaging and histopathological features to the present research on oral and sinonasal SCC and demonstrate the need for further studies with larger populations to investigate the heterogeneous biological behaviour of equine and feline HNSCC. Thus, gaining more specific knowledge regarding the molecular genetics and biology of SCC may help clarify the role of veterinary models in human research and help target specific therapies and redefine prognoses in animals and potentially in humans. Finally, as veterinary and human medicine get more complementary, they might benefit from one another by serving the same goal of one common health.

## Data Availability Statement

The raw data supporting the conclusions of this article will be made available by the authors, without undue reservation.

## Ethics Statement

Ethical review and approval was not required for the animal study because the study is retrospective in nature and the study patients were clinical patients of the Vetmeduni Vienna. Written informed consent for participation was not obtained from the owners because the majority of patient owners signed a consent procedure for research when hospitalizing their patients. Due to the retrospective nature of the study, a minority of patient owners (3/23) agreed to a clinical investigation and were not aware of a subsequent systematic analysis.

## Author Contributions

All authors listed have made a substantial, direct and intellectual contribution to the work, and approved it for publication.

## Conflict of Interest

The authors declare that the research was conducted in the absence of any commercial or financial relationships that could be construed as a potential conflict of interest.
